# Efficacy of Topical Bevacizumab in High-Risk Corneal Transplant Survival

**DOI:** 10.12669/pjms.292.3089

**Published:** 2013-04

**Authors:** Nasir Bhatti, Umair Qidwai, Munawar Hussain, Asif Kazi

**Affiliations:** 1 Nasir Bhatti, Isra Postgraduate Institute of Ophthalmology and Yasin Eye Hospital, Karachi, Pakistan.; 2 Umair Qidwai, Isra Postgraduate Institute of Ophthalmology and Yasin Eye Hospital, Karachi, Pakistan.; 3 Munawar Hussain, Isra Postgraduate Institute of Ophthalmology and Yasin Eye Hospital, Karachi, Pakistan.; 4 Asif Kazi, Isra Postgraduate Institute of Ophthalmology and Yasin Eye Hospital, Karachi, Pakistan.

**Keywords:** Corneal neovascularization, Topical

## Abstract

***Objective: ***To evaluate the effectiveness of topical Bevacizumab in preventing neovascularisation on high risk corneal grafts.

***Methodology: ***This study was a randomized, controlled, parallel group study, carried out from February 2008 to April 2012 (51 months) at Isra Postgraduate Institute of Ophthalmology and Yasin eye hospital, Karachi. Eyes with high risk corneal transplantation with corneal neovascularization were included in this interventional study/randomized clinical trial. Patients were randomly allocated to 2 groups. Group A and Group B. After penetrating keratoplasty, group A patients received topical bevacizumab (2.5%, 25 mg/ml) which was self-administered 4 times a day for 24 week while group B patients received only sham eye drops. Group B was the control group. Corneal neovascular invasion area i.e. the fraction of area on transplanted cornea in which vessels were present was measured using mathematical software program MatLab. Data analyses was done using SPSS version 19. Frequencies of age gender and groups were measured. Neovascular invasion area and change in visual acuity was compared between the 2 groups using *paired t test. P value of less than* 0.05 was considered significant.

***Results: ***Among the 2 groups mean Corneal neo vascular invasion area was minimum in group A (6.23%) while in group B it was (26.7%). Maximum number of patients (26) attained visual acuity of 6/36 or better in the topical bevacizumab group compared to 17 sham group.

***Conclusion: ***When topical Bevacizumab is used, it reduces the recurrence of neovascularisation and thus helps increasing the frequency of graft survival in cases of high risk corneal transplants.

## INTRODUCTION

Corneal graft failure is one of the most common causes of performing keratoplasty again.^1^ When vessels are present on the cornea then chances of graft rejection are very high, and it is considered as a high risk corneal graft surgery.^2^ This high rate of corneal graft failure in cases of vascularized corneas is because after corneal graft these patient can still develop severe immune reaction against the graft and even immunosuppressive therapy cannot help much in the graft survival in such cases.^3^

Many investigations have been carried out to modulate this immune reaction in order to improve keratoplasty results, including prevention of further angiogenesis.^4^^,^^5^ Many molecular factors have been identified in different studies, that can be important elements in angiogenesis.^6^ One of the factors identified related to neovascularization is Vascular endothelial growth factor (VEGF).^4^ Many VEGF inhibitors have been identified and are used for many retinal disorders,^7^ one of such VEGEF inhibitor is Bevacizumab.

Considering the effectiveness of Bevacizumab as an anti VEGF, it has been investigated as a possible treatment option for neovascularization on cornea.^8^^-^^13^ One study carried out on animals, showed that, when bevacizumab was injected systemically in animals having vascularized corneas before doing keratoplasty, it resulted in improved survival of corneal graft.^4^ After these studies it can be assumed that bevacizumab when used either topically or subconunctivally might result in increasing the survival of the corneal graft after keratoplastyby inhibiting the vascularization on the grafted cornea. Considering these facts, we wanted to assess the effectiveness of bevacizubamin reducing the neovascularization thus resulting in better prognosis after high risk. We used a quantitave method of assessing the corneal neovascularization on grafted cornea after keratoplasty by measuring the invasion area of neovessels.

## METHODOLOGY

This study was an interventional/randomized, controlled, parallel group study which was carried out from February 2008 to April 2012 (51 months) at Isra Postgraduate Institute of Ophthalmology and Yasin eye hospital, Karachi. Patients were recruited according to inclusion criteria for the study from February 2008 to September 2011; all the patients were followed up for 6 months after the procedure. Eyes with high risk corneal transplantation with corneal neovascularization were included in this randomized clinical trial using probability purposive sampling. Sample size was not calculated but was dependent on the incident of the patients appearing in the hospital with inclusion and exclusion criteria. Ethical approval was taken from the ethical committee of Isra Postgraduate Institute of Ophthalmology. Informed written consent was taken from every patient included in the study. Indications for PKP were: vascularized leucomas after herpetic keratitis, traumatic keratitis or chemical burn, advanced pseudophakic bullous keratopathy with superficial and deep corneal vascularization, severe infection in hereditary corneal dystrophy, and failed corneal grafts. Patients were randomly allocated to 2 groups. Group A and Group B. After penetrating keratoplasty, group A patients received topical bevacizumab (2.5%, 25 mg/ml) which was self-administered 4 times a day for 24 week while group B patients received only sham eye drops. Group B was the control group. Neither subjects nor investigators were masked, but those who tested visual acuity, optometrists and statistical analysers were masked as regards treatment assignment of the eyes. Before undergoing procedure baseline data was recorded in a proforma. Follow-up period was 2 to 8 months (mean 7.1 months). In order to reduce effect modifiers, during this period all the patients were exposed to similar topical and systemic medications such as systemic steroids and topical steroids according to the weight adjustments. Patients were asked to follow up every 4 weeks from the first postoperative day. On every follow up digital corneal photograph was taken along with the visual acuity. Patients not keeping up with follow ups were excluded from the study in order to exclude the possible confounding variables.

The primary outcome variable was corneal neovascular invasion area while secondary variable was change in visual acuity. In order to do the assessment of neovessels on the grafted cornea, a quantitave method is used. We first took the photograph of the corneas after keratoplasty and then we processed these photographs using different software such as Photoshop and MatLab. By using these software we identified the neovessels on the grafted corneas and measured the area covered by the neovessels on the grafted corneal. This neovascularised area is calculated as percentage of grafted cornea covered by neoveseles. Data analyses were done using SPSS version 19. Frequencies of age gender and groups were measured. Primary variable, neovascular invasion area, was compared between the 3 groups using paired t test. While secondary variable, change in visual acuity, was also compared between the groups using paired t test. P value of less than 0.05 was considered significant.

## RESULTS

Eighty one Patients were included in the study, of them 40 were in the group A (topical bevacizumab), 41 in Group B (sham) Out of these 81 patients, 62 (76.5%) were males while 19 (23.5%) were females. Mean age of the patients was 52.07 (standard deviation=5.54) with minimum age of 39 years and maximum age of patients was 67 years.

Among the 2 groups mean corneal neovascular invasion area was minimum in the topical bevacizumabgroup (group A), with p value = 0.03 (Table-I). Maximum number of patients 26 (65%) attained visual acuity of 6/36 or better in the topical bevacizumab group compared to 17 (41.5%).(p=0.04).(Table-II)

**Table-I:** Corneal neovascular invasion area among the two groups

	*Week 4*	*Week 8*	*Week 12*	*Week 24*
Group A (Bevacizumab )	12.5	9.7	10.5	9.3
Group B (Control)	14.2	18.3	19.8	26.7

P =0.03

**Table-II:** Comparison of visual outcome between the groups

	*6/6-6/18*	*6/24-6/36*	*6/60-3/60*	*2/60-HM*
Group A (Bevacizumab )	12 (30%)	14 (35%)	10 (25%)	5 (12.5%)
Group B (Control)	6 (14.6%)	11 (26.8%)	18 (43.9%)	6 (14.6%)

P=0.04

## DISCUSSION

Bevacizumab has been the topic of study by many researcher all over the world in ophthalmology whenever they are coping with neovascularization either in the form of neovascularization on retina or neovascularization on cornea. One study by Bock F showed that topical applications of bevacizumab significantly inhibited the outgrowth of blood (P < 0.0001) and lymphatic (P < 0.0001) vessels. Inhibition of the proliferation of leukocytes was also significant (P < 0.0001).^14^ He also showed that, Western blot analysis, ELISA, and the surface Plasmon resonance assay showed that bevacizumab binds murine VEGF-A. thus they concluded that topical application of bevacizumab inhibits both inflammation-induced angiogenesis and lymph angiogenesis in the cornea.^14^ The findings in his study suggests an important role of VEGF-A in corneal lymph angiogenesis.^14^ He also concluded that Bevacizumab may be useful in preventing immune rejections after penetrating keratoplasty or tumor metastasis via lymphatic vessels. Many researchers have applied this knowledge in reducing the corneal neovascularization.^15^ One such study was also performed by us in our local setup.^15^ We applied Bevacizumab in patients with corneal neovascularization subconjunctivally but failed to see statistically significant improvement in neovascularization.^15^ But many studies performed internationally have shown promising results when bevacizumab is applied in order to reduce the corneal neovascularization.^16^^-^^18^

Our study showed that bevacizumab when used topically resulted in reduction of neovascularization on the grafted cornea and thus improve the survival of corneal graft after keratoplasty. No such study was performed on grafted cornea using topical bevacizumab locally/Nationally so we have compared the results of our study with International studies performed on topical bevacizumab on grafted cornea. Most of the work has been done on the subconjunctival route of bevacizumab rather than the topical route so very little work has been done globally of topical route of bavacizumab use for high risk grafts. One study by Dastjerdi , showed that the mean percentage reduction of neovascular area was 33% (P = 0.15) when topical bevacizumab was used for high risk corneal transplants compared to control group.^16^

The main limitations of the study were that it was conducted in only 2 centers thus not much of the variability was available in terms of patients was concerned. Both the centers were located in the urban city dealing mostly with population of similar ethnic background.

## CONCLUSION

Bevacizumab when given topically can help in increasing the frequency of graft survival in cases of high risk corneal transplants. More studies are needed to further strengthen these conclusions.
